# Sexual dysfunction among Ghanaian men presenting with various medical conditions

**DOI:** 10.1186/1477-7827-8-118

**Published:** 2010-10-13

**Authors:** Nafiu Amidu, William KBA Owiredu, Eric Woode, Roselyn Appiah, Lawrence Quaye, Christian K Gyasi-Sarpong

**Affiliations:** 1 Department of Medical Laboratory Technology, Faculty of Allied Health Sciences, College of Health Sciences, Kwame Nkrumah University of Science and Technology, Kumasi, Ghana; 2 Department of Molecular Medicine, School of Medical Sciences, College of Health Sciences, Kwame Nkrumah University of Science and Technology, Kumasi, Ghana; 3 Department of Pharmacology, Faculty of Pharmacy and Pharmaceutical Science, College of Health Sciences, Kwame Nkrumah University of Science and Technology, Kumasi, Ghana; 4 Department of Surgery, (Urology Unit) Komfo Anokye Teaching Hospital/College of Health Sciences, Kwame Nkrumah University of Science and Technology, Kumasi, Ghana

## Abstract

**Background:**

Several medical conditions can affect and disrupt human sexuality. The alteration of sexuality in these medical conditions often hinder effective communication and empathy between the patients and their sexual partners because of cultural attitudes, social norms and negative feelings such as anxiety and guilt. Validated and standardized sexual inventories might therefore help resolve this problem. The objective of this cross-sectional study was to obtain data on the prevalence of male sexual dysfunction (SD) among Ghanaians with various medical conditions residing in Kumasi.

**Methods:**

The Golombok Rust Inventory of Sexual Satisfaction (GRISS) was administered to 150 Ghanaian men with various medical conditions between 19 and 66 years old (mean ± standard deviation: 40.01 ± 12.32 years) domiciled in the Kumasi metropolis.

**Results:**

Out of the total 150 questionnaires administered, 105 (70.0%) men returned the questionnaires. Questionnaires from 3 men were incomplete, leaving 102 complete and evaluable questionnaires, indicating a 68.0% response rate. Of the remaining 102 men, 88.2% were married, 70.6% had attained higher education, 88.2% were non-smokers. Whereas 54.9% were engaged in exercise, 61.8% indulged in alcoholic beverages. The prevalence of the various medical conditions include: diabetes (18%), hypertension (24.5%), migraine (11.8%), ulcer (7.8%), surgery (6.9%), STD (3.9) and others (26.5%). The prevalence of SD among the respondents in the study was 59.8%. The highest prevalence of SD was seen among ulcer patients (100%), followed by patients who have undergone surgery (75%), diabetes (70%), hypertension (50%), STD (50%) and the lowest was seen among migraine patients (41.7%).

**Conclusions:**

SD rate is high among Ghanaian men with medical conditions (about 60%) and vary according to the condition and age.

## Background

Sexual dysfunction (SD) is an important public health problem that compromises the overall quality of life of the patients and their sexual partners [1,2]. This consequently leads to loss of emotional and physical intimacy and at times leads to divorce. Customarily, male SD has been attributed to psychogenic factors, however, advances in pathophysiology research indicate vascular malfunction in the majority of patients. The vascular malfunction could be as a result of atherosclerotic lesions in the penile arteries that consequently lead to diminished blood flow.

About 322 million men worldwide are projected to develop erectile dysfunction (ED) by the year 2025 with the largest projection increases in the developing world that is Africa, Asia, and South America. Africa is projected to have the highest percentage increase of 169% from 1995 to 2025 [3]. The variation in the prevalence of SD ranges from 15% in Brazil to 74% in Finland depending upon the methodology, target group, medical conditions, sample size, the definition of SD used as well as cultural and religious differences [4-9]. Data from some African, Arabic, or Islamic countries with similar socio-cultural and religious characteristics to Ghana indicate SD prevalence of 54.9% in Egypt, 50.7% in Nigeria and 64.3% in Turkey [10]. Previous study by our group among the general Ghanaian populace indicates 66% prevalence of SD [11].

Earlier studies have demonstrated an association between SD and medical conditions such as vascular disease [12,13] and cardiovascular risk factors [13]. SD could be a symptom and/or a marker of vascular disease progression [14]. Hypertension is one of the common medical conditions [15], together with diabetes mellitus, migraine, ulcer, surgery, sexual transmitted diseases and dyslipidaemia that could modify the sexual function of an individual. Even though, most of these are widely accepted as risk factors for SD, available data are controversial and indicate that this relationship is not well established. For example, the reports of Virag *et al.,*[16], Shabsigh *et al.,*[17] and Jaffe *et al.,*[18] indicated that hypertension was not an independent predictor of vasculogenic ED. The objective of this cross-sectional study was to obtain data on the prevalence of male SD among Ghanaian men presenting with various medical conditions residing in Kumasi. To our knowledge, this is the first study of SD conducted among this population in Ghana.

## Methods

### Subjects

This epidemiological cross-sectional study was conducted among subjects with various medical conditions in the Kumasi metropolis, Ghana between January and April 2010. All the participants were sexually active Ghanaians, aged 19 years and above, who had maintained a stable heterosexual relationship for at least 2 years before enrollment in the study. A stable relationship was defined as one in which the man maintains sexual relations regardless of marital status. A random method was used to administer the questionnaires to a total of 150 heterosexual men with various medical conditions within the Kumasi Metropolis. Ulcer as used in this study includes any form of stomach ulcer and surgery involved any form of surgery. Participation of the respondents was voluntary and informed consent was obtained from each participant. The study was approved by the Committee on Human Research, Publication and Ethics of the School of Medical Science and the Komfo Anokye Teaching Hospital, Kumasi.

### Questionnaires

Sexual response was measured by the Golombok Rust Inventory of Sexual Satisfaction (GRISS) questionnaire. The GRISS has 28 items on a single sheet and it is used for assessing the existence and severity of sexual problems. All the 28 questions are answered on a five-point scale from "always", through "usually', "sometimes", and "hardly ever", to "never". It gives overall SD scores and also gives a profile for the men on 7 subscales, comprising impotence, premature ejaculation, infrequency, non-communication, non-sensuality, avoidance and dissatisfaction. Responses are summed up to give a total raw score (range 28-140). The total score and subscale scores are transformed using a standard nine point scale, with high scores indicating greater problems. Scores of five or more are considered to indicate SD. The GRISS was chosen because it is standardized, easy to administer and score, relatively unobtrusive and substantially inexpensive. The reliability of the overall scales has been found to be 0.94 for men, and that of the subscales on average 0.74 (ranging between 0.61 and 0.83). Validity has been demonstrated under a variety of circumstances [19-21].

### Statistical analysis

The data were presented as mean ± standard deviation or percentages. Continuous data were analyzed using unpaired *t*-tests whilst categorical variables were analyzed using Fischer's exact tests. In all statistical tests, a value of *p* < 0.05 was considered significant. All analyses were performed using SigmaPlot for Windows, Version 11.0, (Systat Software, Inc. Erkrath, Germany) [22].

## Results

Out of the total 150 questionnaires administered, 105 (70.0%) men returned the questionnaires. The questionnaires from 3 men were incomplete, leaving 102 complete and evaluable questionnaires, indicating a 68.0% response rate. The age range for the responding men was 19 to 66 years with a mean ± standard deviation of the age being 40.01 ± 12.32 years. Majority of the men who responded were married (88.2%), had attained higher education (70.6%), consumed alcoholic beverages (61.8%) and are non-smokers (88.2%). About half of the study population were engaged in exercise (54.9%). The respondents suffered from the following medical conditions: diabetes (18%), hypertension (24.5%), migraine (11.8%), ulcer (7.8%), surgery (6.9%), STD (3.9%) and others (26.5%). When the respondents were stratified based on sexual function, those with SD were older, married, had a longer duration of marriage and performed little or no exercise as compared to those without SD as shown in Table 1. The overall GRISS score and the score for each subscale were significantly higher in responding men with SD as compared to without SD (Table 2).

**Table 1:** General characteristic of the studied population stratified by SD

Variables	Total (102)	Without SD (41)	With SD (61)	P value
Age (yrs)	42.01 ± 12.32	37.32 ± 11.26	45.16 ± 12.08	0.0013
Married (%)	90(88.2)	33(80.5)	57(93.4)	0.0465
Duration of marriage (yrs)	12.99 ± 9.8	10.29 ± 8.5	14.61 ± 10.2	0.0413
High education (%)	72(70.6)	29(70.7)	43(70.5)	0.9792
***Cigarette smoke per years***				
0	90(88.2)	35(85.4)	55(90.2)	0.4609
< 10	9(8.8)	5(12.2)	4(6.6)	0.3250
10-20	3(2.9)	1(2.4)	2(3.3)	0.8056
> 20	0(0.0)	0(0.0)	0(0.0)	
***No of big bottle of alcohol taken per week***				
0	39(38.2)	14(34.1)	25(40.9)	0.4860
< 10	53(52.0)	23(56.1)	30(49.2)	0.4930
10-20	10(9.8)	4(9.8)	6(9.8)	0.9894
> 20	0(0.0)	0(0.0)	0(0.0)	
***No of exercise per week***				
0	45(44.1)	13(31.7)	32(52.5)	0.0385
weekends only	42(41.2)	19(46.3)	23(37.7)	0.3849
1-5 times	9(8.8)	4(9.8)	5(8.2)	0.7854
> 5 times	5(4.9)	4(9.8)	1(1.6)	0.0627
***Medical conditions***				
Diabetes	19(18.6)	2(4.9)	17(27.9)	0.0035
Hypertension	25(24.5)	9(22.0)	16(26.2)	0.6224
Migraine	12(11.8)	5(12.2)	7(11.5)	0.9119
Ulcer	8(7.8)	1(2.4)	7(11.5)	0.0960
Surgery	7(6.9)	3(7.3)	4(6.6)	0.8817
STD	4(3.9)	2(4.9)	2(3.3)	0.6833
Others	27(26.5)	17(41.5)	10(16.4)	0.0049

Continuous data are presented as mean ± standard deviation and categorical data presented as proportion. Continuous data were compared using unpaired t-test and categorical data compared using chi square analysis.

**Table 2:** Raw score as well as stannine score for the various GRISS subscales stratified by SD.

Variables	Total (n = 102)	Without SD (n = 41)	With SD (n = 61)	P value
***Raw score for the various GRISS subscales***				
Impotence	11.5 ± 2.4	10.1 ± 2.3	12.5 ± 1.9	0.0016
infrequency	6.1 ± 1.2	5.6 ± 1.2	6.5 ± 1.1	0.0201
Non-communication	5.6 ± 1.5	5.0 ± 1.6	6.0 ± 1.4	0.0474
Dissatisfaction	11.2 ± 2.3	9.7 ± 2.3	12.2 ± 1.6	0.0008
Avoidance	8.2 ± 3.2	6.9 ± 2.7	9.4 ± 3.3	0.0229
Non-sensuality	11.7 ± 2.3	10.6 ± 2.5	12.4 ± 1.9	0.0195
Premature ejaculation	9.6 ± 2.5	8.1 ± 2.1	10.6 ± 2.2	0.0009
***Stannine score for the various GRISS subscales***				
Impotence	5.0 ± 1.7	4.0 ± 1.7	5.6 ± 1.4	0.0019
infrequency	5.0 ± 1.7	4.2 ± 1.7	5.4 ± 1.6	0.0224
Non-communication	5.2 ± 1.9	4.4 ± 2.0	5.7 ± 1.7	0.0294
Dissatisfaction	5.1 ± 1.9	3.9 ± 1.9	5.1 ± 1.4	0.0005
Avoidance	5.0 ± 2.0	4.1 ± 1.9	5.5 ± 1.9	0.0177
Non-sensuality	4.9 ± 1.8	4.3 ± 1.8	5.4 ± 1.6	0.0601
Premature ejaculation	5.0 ± 1.9	3.9 ± 1.6	5.8 ± 1.6	0.0010

Results are presented as mean ± Standard Deviation, SD = SD.

**Figure 1:**
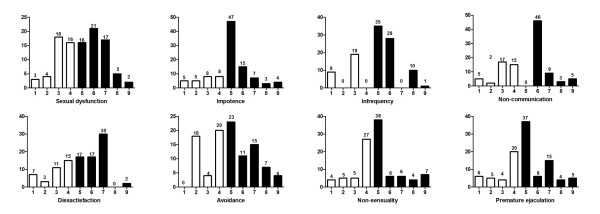
**Scores of sexual dysfunction in 102 studied patients according to GRISS questionnaire**. Graph shows the distribution of scores (from 1 to 9 on the x- axis) for each GRISS subscale, with the number of patients (y-axis) above each score. Normal scores range from 1-4 and abnormal scores are 5-9.

The prevalence of SD among the respondents in the study was 59.8% (61 out of 102) as shown in Figure 1. The most prevalent areas of difficulty were impotence (76 out of 102, 74.5%), infrequency (74 out of 102, 72.5%), pre-mature ejaculation (67 out of 102, 65.7%), dissatisfaction (66 out of 102, 64.7%), non-communication (63 out of 102, 61.8%), non-sensuality (61 out of 102, 59.8%), avoidance (60 out of 102, 58.8%) (Figure 1).

The likelihood of being sexually active declined steadily with age. When the study population was stratified based on age, individuals with difficulties with SD, impotence, non-communication and infrequency gave a significant trend with age using Chi square for trend analysis as indicated in Table 3. The proportion of individuals with severe difficulties did not give any significant trend with age (Table 3).

**Table 3:** Prevalence of SD stratified by age among the studied population

Variables	Age (years)							P value
	18-22	23-27	28-32	33-37	38-42	43-47	> 48	
***n***	***6***	***5***	***18***	***13***	***13***	***13***	***34***	
	Difficulties (%)*							
SD	16.7	0.0	66.7	53.9	53.9	46.2	79.4	0.0021
Impotence	50.0	20.0	72.2	76.9	76.9	69.2	88.2	0.0022
Infrequency	66.7	20.0	72.2	76.9	69.2	53.9	85.3	0.0808
Non-Communication	16.7	33.3	44.4	76.9	76.9	53.8	73.5	0.0071
Dissatisfaction	33.3	16.7	50.0	76.9	61.5	53.8	85.3	0.7803
Avoidance	50.0	60.0	55.6	46.1	46.1	53.8	73.5	0.1383
Non-sensuality	33.3	40.0	72.2	76.9	76.9	61.5	47.1	0.5401
Premature ejaculation	66.7	40.0	88.9	69.2	76.9	46.2	58.8	0.1771
	Severe difficulties(%)**							
SD	0.0	0.0	11.1	15.4	15.4	0.0	2.9	0.4719
Impotence	0.0	0.0	16.7	7.7	7.7	0.0	5.9	0.5094
Infrequency	16.7	0.0	16.7	0.0	15.4	0.0	14.7	0.8999
Non-Communication	0.0	0.0	11.1	23.1	0.0	7.7	5.9	0.7803
Dissatisfaction	0.0	0.0	0.0	15.4	0.0	0.0	0.0	0.4875
Avoidance	0.0	20.0	27.8	7.7	15.4	7.7	2.9	0.0879
Non-sensuality	16.7	0.0	22.2	7.7	0.0	15.4	8.8	0.4795
Premature ejaculation	0.0	0.0	22.2	7.7	15.4	0.0	5.9	0.4247

*Score of 5-9 and **Score of 8 or 9

As shown in Table 4, whereas the highest prevalence of individuals with difficulties with SD (100.0%), impotence (100.0%), infrequency (88.9%), non-communication (100.0%), dissatisfaction (88.9%) and avoidance (88.9%) were seen in ulcer patients, the highest prevalence of non-sensuality (70.0%) and premature ejaculation (80.0%) were seen in diabetics. However, the lowest prevalence of SD (41.7%), non-communication (41.7%) and avoidance (25.0%) were seen in individuals with migraine, the lowest prevalence of impotence (50.0%), non-sensuality (50.0%) and premature ejaculation (50.0%) were seen among individuals with STD and that of infrequency (63.6%) and dissatisfaction (59.1%) were seen among hypertensive patients (Table 4).

**Table 4:** Prevalence of SD stratified by age among the studied population

Variables	Diabetes	Hypertension	Migraine	Ulcer	Surgery	STD	Others
***n***	***20***	***22***	***12***	***9***	***8***	***4***	***27***
	Difficulties (%)*						
SD	70.0	50.0	41.7	100.0	75.0	50.0	51.9
Impotence	75.0	68.2	75.0	100.0	87.5	50.0	63.0
Infrequency	80.0	63.6	75.0	88.9	75.0	75.0	66.7
Non-Communication	60.0	54.5	41.7	100.0	50.0	75.0	66.7
Dissatisfaction	70.0	59.1	66.7	88.9	62.5	75.0	55.6
Avoidance	85.0	50.0	25.0	88.9	62.5	25.0	55.6
Non-Sensuality	70.0	59.1	66.7	66.7	62.5	50.0	48.1
Premature Ejaculation	80.0	59.1	75.0	66.7	62.5	50.0	59.3
	Severe difficulties (%)**						
SD	20.0	4.5	0.0	22.2	0.0	0.0	0.0
Impotence	5.0	13.6	0.0	22.2	0.0	0.0	3.7
Infrequency	20.0	9.1	0.0	11.1	12.5	0.0	11.1
Non-Communication	5.0	18.2	0.0	11.1	0.0	25.0	3.7
Dissatisfaction	0.0	0.0	8.3	11.1	0.0	0.0	0.0
Avoidance	30.0	9.1	0.0	0.0	0.0	0.0	11.1
Non-Sensuality	20.0	13.6	0.0	0.0	25.0	25.0	3.7
Premature Ejaculation	0.0	9.1	8.3	11.1	12.5	25.0	14.8

*Score of 5-9 and **Score of 8 or 9.

Generally, the proportion of individuals with severe difficulties with SD as well as its subscale was highest among individual with ulcer followed by diabetes and hypertension. The lowest prevalence was however seen among individuals with migraine followed by those who have undergone surgery and STD (Table 4).

From Table 5, age correlated positively with duration of marriage, SD, infrequency, non-communication, and dissatisfaction. Socio-demographic characteristics (i.e. duration of marriage, education, smoking, alcohol and exercise) did not give any significant association with SD as well as its subscales. However, SD correlated positively with medium to large size effect with its subscales and the subscales also generally correlated positively with each other with small to medium size effect as shown in Table 5.

**Table 5:** Pearson product moment correlation coefficient between SD including the 7 subscales of the GRISS (N = 102)

Variables	DUR	EDU	SMK	ALC	EXR	SD	IMP	IFQ	NC	DISS	AVD	NS	PE
Age	0.90***	0.09	-0.02	-0.05	0.14	0.29**	0.17	0.25*	0.24*	0.28**	-0.01	-0.04	0.03
Duration (DUR)		0.17	0.00	-0.10	0.20	0.15	0.04	0.19	0.14	0.16	0.01	-0.07	0.01
Educational level (EDU)			0.24*	0.05	0.00	0.03	0.02	0.00	0.01	0.12	-0.07	0.02	-0.05
Smoking (SMK)				0.16	0.28**	-0.03	0.03	0.08	-0.16	-0.02	-0.05	-0.12	-0.03
Alcohol consumption(ALC)					0.28**	0.08	0.16	0.05	-0.07	-0.08	0.19	-0.02	-0.12
Exercise (EXR)						0.14	0.17**	0.17**	0.01	0.04	0.11	-0.05	0.12
SD (SD)							0.67***	0.48***	0.43***	0.57***	0.50***	0.50***	0.62***
Impotence (IMP)								0.23**	0.17**	0.20*	0.30***	0.28***	0.44***
Infrequency (IFQ)									0.07	0.28**	0.12	0.15	0.36***
Non-communication (NC)										0.26**	0.00	0.09	0.23*
Dissatisfaction (DISS)											0.10	0.16*	0.18*
Avoidance (AVD)												0.11	0.38***
Non-sensuality (NS)													0.19*

*Correlation is significant at the 0.05 level (2-tailed), **Correlation is significant at the 0.01 level (2- tailed),***Correlation is significant at the 0.001level (2-tailed). Boldface r = Pearson product moment correlation coefficient with a medium size (0.30 ≤ r ≥ 0.50) effect: boldface and underline r = Pearson product moment correlation coefficient with a large size(r > 0.50) effect, PE = Premature ejaculation.

## Discussion

Comprehensive reviews of sexuality, psychopathology and epidemiological literature suggest that sexual problems occur frequently [23] and despite increasing demand for clinical services and the potential impact of disorders of SD on interpersonal relationships and quality of life [24,25], epidemiologic data are relatively scanty.

SD is said to be common among men of all ages with a prevalence that varies from 15% to 74% depending upon the methodology, target group, sample size, and the definition of SD used [4-9]. Besides, the influence of the underlying medical conditions as well as cultural, religious and perceptual differences on SD is not known. Whereas the observed prevalence rate of 59.8% from this cohort is in agreement with available data on prevalence range of SD (i.e. 15% to 74%), it is slightly higher than data from African countries with similar sociocultural and religious characteristics (i.e. 54.9% in Egypt, and 50.7% in Nigeria (Pfizer)). This prevalence rate is however close to the 64.3% prevalence rate from Turkey [10] but lower than the 66% prevalence rate reported earlier among the general male populace from the same city in Ghana by Amidu *et al.,*[11].

These variations could be largely due to difference in methodology, sample size, definition of SD, inherent standard and belief of an individual, most importantly, underlying medical conditions and perceptual differences. Ghanaians in this part of the country are known to perceive an intravaginal ejaculatory latency of 7-25 min as being normal, with about 75% perceiving adequate intravaginal ejaculatory latency time above what sex therapists perceived as being adequate (i.e. 3-7 min). All these are also modified by the type of formal and informal education received from the society (Amidu *et al.,* under review).

Men with SD in this study were significantly older, married with longer duration of marriage and are most likely not to engage in any exercise when compared to males without SD. Laumann *et al.,*[4] in a study on SD in the United States reported that older men are more likely to have trouble maintaining or achieving an erection as well as to lack an interest in sex and attributed it to the physiological changes associated with the aging process. Likewise, data from the Massachusetts Male Aging Study (MMAS) also showed that 34.8% of men aged 40 to 70 years had moderate to complete ED which was strongly related to age and health status [12]. Frank *et al.,*[26] also identified SD among couples believed to have a stable relationship with 7% of men reporting difficulties in achieving and 9% in maintaining an erection.

Advocates of exercise claim that physical activity may enhance sexual performance and sexual pleasure [27,28]. Physical endurance, muscle tone and body composition all improve sexual functioning [27] and according to literature, sedentary men could significantly lower their risk of SD by burning at least 200 calories per day (equal to fast-walking for about 2 miles) [28]. Bacon *et al.,*[8] in a research to check which lifestyle factors affected the risk of SD observed that, men over 50 years who kept physically active had a 30% lower risk of SD compared with inactive men. The findings of this study are consistent with that of Bacon *et al* which emphasized the role of age and lack of physical inactivity in the development of SD and the existence of SD among married men.

A further observation of a high prevalence of SD (79.4%) in men > 48 years with impotence (88.2%) and non-communication (73.5%) being the subscale areas of difficulty agrees with previous studies [4,8,12]. Furlow [29] defined impotence as the persistent failure to develop erections of sufficient rigidity for penetrative sexual intercourse and further reported it as being strongly related to age, with an estimated prevalence of 2% at age 40 years which rises to 25 to 30% by the age of 65. The high prevalence of impotence observed in this study shows that impotence is an inherent public health problem which will need rapt medical attention considering the fact that a high percentage of males with this disorder will most likely not communicate about it thereby affecting interactions with family and associates. Impotence showed positive correlations with all the other subscales of GRISS, correlating with avoidance and premature ejaculation to a medium size effect depicting a likelihood of avoidance of sexual activities in males found in this group. This finding is in agreement with the study of Tsitouras *et al.,*[30], who reported a progressive decline in the frequency of sexual intercourse with advancing age when men between the ages of 60 and 79 were examined in the Baltimore Aging Study.

Diseases may greatly alter the sexuality of an individual and many systemic diseases reduce testosterone leading to a decrease in libido [31]. Enzlin *et al.,*[32] in a study on the prevalence and predictors of SD in patients with type 1 diabetes reported a SD prevalence rate of 22% in diabetic males and 40.5% in males with diabetic complications. Twenty percent (20%) of diabetic males in this study had severe difficulties with SD which compares well with the findings of Enzlin *et al.,*[32] whilst 70% had difficulties with SD, a prevalence rate which is higher than that reported in available literature. McCulloch *et al.,*[33] in a survey of diabetic males, aged 20 to 59 years reported an impotence prevalence rate of 35% and Furlow [29] estimated an impotence prevalence rate of 35 to 50% in diabetic men. The impotence prevalence rate (5%) in diabetic males with severe difficulties is lower compared to observed rates in the studies of Furlow [29] and McCulloch *et al.,*[33] whilst the impotence prevalence rate of 75% estimated in diabetic males with difficulties in SD is higher than reported in available literature.

Several reports have indicated that 2.4 to 58% of hypertensive males experience one or more symptoms of SD of varying degrees of severity [34,35]. Fifty percent (50%) of hypertensive males had difficulties with SD and 4.5% had severe difficulties with SD which agrees with prevalence rates quoted in available literature. Jensen *et al.,*[36] in their study on the prevalence and etiology of impotence in male hypertensive outpatients reported a prevalence rate of 27%. An impotence prevalence rate of 68.2% and 13.6% was estimated in male hypertensives with difficulties and severe difficulties in SD respectively.

Higher sexual desire inventory scores have been reported in subjects with migraine [37,38]. Intriguingly, the least prevalence of difficulties with SD (41.7%), non-communication (41.7%) and avoidance of sexual activity (25.0%) was observed in males with migraine in agreement with previous studies. Ironically, the prevalence of impotence in males with migraine was 75% revealing the existence of sexual disorders in migraineurs contrary to reports of increases in sexual desire and this finding is in agreement with the study of [39] who reported that headache contributes to less sexual activity.

The prevalence of SD in males with ulcer, surgery and sexual transmitted disease (STD) was 100%, 75% and 50% respectively with the corresponding impotence prevalence rates of 100%, 87.5% and 50% respectively. SD has been reported in patients who have undergone surgery with surgical procedure being independently associated with current sexual activity [40,41]. Hendren *et al.,*[40] further reported impotence and partial impotence prevalence rates of 32% and 52% respectively in males who underwent surgery for rectal cancer. Filiberti *et al.,*[42] attributed impotency as a consequence of rectal cancer to parasympathetic nerve injury. Fass *et al.,*[43] reported a self-reported SD prevalence rate of 43.3% in patients with functional gastrointestinal (GI) disorders with decreased sexual drive (36.2%) being the common symptom in males and further found SD to be positively associated with perceived GI symptom severity. Ulcer patients in this study had the highest prevalence of SD and a high prevalence for five out of the seven subscale disorders. The reason for the highest prevalence of SD among patients with ulcer is not readily known from this study, thus further studies are needed to elucidate the mechanism.

The observation of significant positive correlations between impotence and the other subscales of GRISS in this study shows the likelihood of co-existence of the other subscales in varying degrees thereby affecting quality of life and relationship between spouses. Some of the limitations of this study include the fact that the study was based on volunteers, includes only male and self-reported data on socio-demographic information as well as the various medical conditions.

## Conclusions

SD rate is high among Ghanaian men with medical conditions (about 60%) and vary according to the condition and age. The highest prevalence of SD was seen among ulcer patients (100%), followed by patients who have undergone surgery (75%), diabetes (70%), hypertension (50%), STD (50%) and the lowest is seen among migraine patients (41.7%).

## Competing interests

The authors declare that they have no competing interests.

## Authors' contributions

NA, WKBAO and EW developed the concept and designed the study. NA, WKBAO, EW, RA, LQ and CKG-S administered the questionnaire, analysed and interpreted the data. NA, RA, LQ, and CKG-S drafted the manuscript. NA, WKBAO, EW, LQ, RA and CKG-S revised the manuscript for intellectual content. All authors read and approved the final manuscript.
